# Characterization of the complete chloroplast genome of *Wisteriopsis reticulata* (Fabaceae): an IRLC legumes

**DOI:** 10.1080/23802359.2022.2079436

**Published:** 2022-06-20

**Authors:** Shanshan Zhu, Aowei Liu, Xiao Xie, Maoqin Xia, Haimin Chen

**Affiliations:** a Systematic & Evolutionary Botany and Biodiversity Group, MOE Laboratory of Biosystem Homeostasis and Protection, College of Life Sciences, Zhejiang University, Hangzhou, Zhejiang, China; b School of Marine Sciences, Ningbo University, Ningbo, Zhejiang, China

**Keywords:** *Wisteriopsis reticulata*, chloroplast genome, IRLC

## Abstract

The inverted repeat‐lacking clade (IRLC) species are characterized by the loss of an IR region in their plastomes, which has long been of great interest. *Wisteriopsis reticulata* is one of the members of the tribe Wisterieae, which belongs to Fabaceae and is well-known as IRLC. Here, we reported and characterized the complete chloroplast genome of *W. reticulata* using the genome skimming approach. The chloroplast genome is 132,477 bp in length and lacks one copy of IR region. The genome encoded 112 unique genes including 89 protein-coding genes, 29 transfer RNA genes and four ribosomal RNA genes. Phylogenetic results supported the monophyly of the tribe Wisterieae (IRLC) and confirmed that *W. reticulata* belongs to the genus *Wisteriopsis*.

The chloroplast genome (cp genome) of angiosperms generally has a conserved quadripartite structure with a large single‐copy (LSC) region and a small single‐copy (SSC) region separated by two inverted repeat (IR) regions (Yurina and Odintsova 1998; Jansen et al. 2005). However, some taxa have lost an IR region in their cp genome, which named the inverted repeat‐lacking clade (IRLC) and included ca. 56 genera and more than 4000 species (Wojciechowski et al., 1999; Wojciechowski et al. 2000; Xia et al. 2021). Wisterieae, belongs to Fabaceae, is well-known as IRLC (Compton et al. 2019). *Wisteriopsis reticulata* (Benth.) Compton & Schrire 2019 is one of the members of the Wisterieae, previously known as *Millettia reticulata* Benth. Pl. Jungh. 1852 and *Callerya reticulata* (Benth.) *Schot* 1994 (Compton et al. 2019). It is a woody climber with racemes of pea-like flowers and serves as a folk Chinese medicine. It is used to treat many kinds of blood disorders and also auto-immune diseases. Given that genetic information is of great significance in species taxonomy, we reported and characterized the complete cp genome of *W. reticulata*, and inferred the phylogenetic relationships of this species.

The fresh leaves of *W. reticulata* were collected from the Zijingang Campus of Zhejiang University (Hangzhou, China, 30°17′55.19″ N, 120°5′6.08″ E). The specimen and DNA sample of this species were deposited at the Herbarium of Zhejiang University (HZJU) under the voucher number HZU0243 (contact person and email: Shanshan Zhu, 21407009@zju.edu.cn). The total genomic DNA was extracted following the CTAB method (Doyle and Doyle 1987) and was then sequenced (paired-end, PE 150 bp) on the BGISEQ-500 platform at Beijing Genomics Institute (Shenzhen, China). Finally, about 3.0 Gb clean data were obtained and assembled into complete cp genome by using the Getorganelle v1.7.4 pipeline (Jin et al. 2020). Gene annotation was performed using the GeSeq (Tillich et al. 2017) and manually checked with the start/stop codons in Geneious v9.0.2 (http://www.geneious.com). The annotated cp genome sequence and the Illumina paired-end sequencing data was registered into the NCBI with the accession number OL022306 and BioProject codes PRJNA783869.

The cp genome of *W. reticulata* was 132,477 bp in length consisting of two single-copy regions (LSC with 87,993 bp; SSC with 19,082 bp) and one repeat region (IR with 25,402 bp). The genome size was much shorter than that of many other angiosperm cp genomes because it has lost one copy of IR region. The GC content of the complete cp genome, LSC, SSC, and IR were 34.2, 32.9, 29.8 and 41.9%, respectively. The genome encoded 112 unique genes including 89 protein-coding genes, 29 tRNA genes and 4 rRNA genes. Ten protein-coding genes and five tRNA genes contained one single intron, whereas two genes (*paf*l, *clp*P) had two introns.

A total of 29 complete cp genomes in Wisterieae (Fabaceae) together with four cp genomes of *Millettia* species were applied to infer the phylogenetic position of *W. reticulata*. *Millettia* species were selected as outgroups. The sequence alignment was conducted with MAFFT v7.407 (Katoh and Standley 2013). The maximum likelihood (ML) analyses were performed using IQ-TREE v. 2.1.2 (Minh, 2020) (Figure 1). The best fitting model was chosen by using MODELFINDER (Kalyaanamoorthy et al. 2017). Support values for each internal branch were evaluated using 1000 ultrafast bootstrap replicates. Our phylogenetic analyses supported the monophyly of the tribe Wisterieae, which is one of the earliest diverged groups of IRLC (Duan et al. 2021). Within Wisterieae, the phylogenetic tree supported five main clades that were largely consistent with previous analyses (Compton et al. 2019; Duan et al. 2021). Noticeably, *Afgekia sericea* did not belong to any clade but was closely related to clades C + D. In addition, our results indicated that *W. reticulata* was the basal taxa within *Wisteriopsis* with 100% bootstrap, which provided stronger evidence for this species taxonomy at complete chloroplast genome level. Namely, *W. reticulata* should belongs to the genus *Wisteriopsis* rather than *Millettia* or *Callerya*. Moreover, the chloroplast genomes resource could be utilized for DNA barcoding, conservation genetics, and breeding of *W. reticulata* in the future.

**Figure 1.**
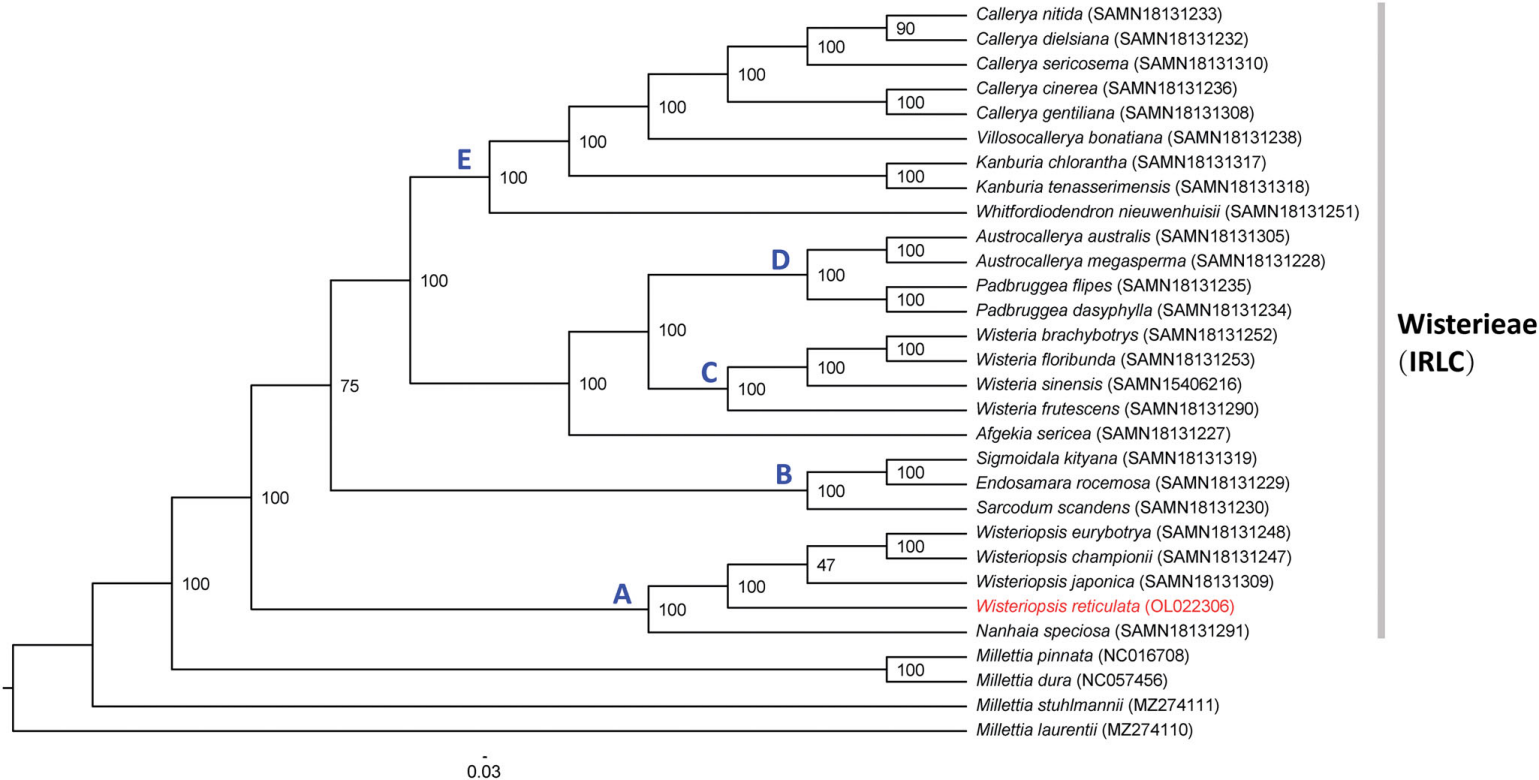
Maximum likelihood phylogenetic tree based on 29 complete chloroplast genome sequences of Wisterieae and four outgroups. Relative branch lengths are indicated. Numbers near the nodes represent ML bootstrap value.

## Ethical approval

The collection of plant material has been carried out in accordance with guidelines provided by Zhejiang University and followed national regulations.

## Author contributions

Chen H. conceived and designed the project. Xia M. collected samples and performed sequencing. Liu A., Xie X. and Zhu S. analyzed and interpretated the data. Zhu S. wrote the manuscript. Chen H. and Xia M. revised the manuscript.

## Data Availability

The genome sequence data that support the findings of this study are openly available in GenBank of NCBI at (https://www.ncbi.nlm.nih.gov/) under the accession no. OL022306. The associated BioProject, SRA, and Bio-Sample numbers are PRJNA783869, SRR17044436, and SAMN23455509, respectively.
